# Moving beyond static snapshots: Protein dynamics and the Protein Data Bank

**DOI:** 10.1016/j.jbc.2021.100749

**Published:** 2021-05-04

**Authors:** Mitchell D. Miller, George N. Phillips

**Affiliations:** 1 Department of Biosciences, Rice University, Houston, Texas, USA; 2 Department of Chemistry, Rice University, Houston, Texas, USA

**Keywords:** protein dynamics, structure function, Protein Data Bank, structural biology, crystallography, electron microscopy, molecular dynamics, nuclear magnetic resonance (NMR), MD, molecular dynamics, PDB, Protein Data Bank, SFX, serial femtosecond crystallography

## Abstract

Proteins are the molecular machines of living systems. Their dynamics are an intrinsic part of their evolutionary selection in carrying out their biological functions. Although the dynamics are more difficult to observe than a static, average structure, we are beginning to observe these dynamics and form sound mechanistic connections between structure, dynamics, and function. This progress is highlighted in case studies from myoglobin and adenylate kinase to the ribosome and molecular motors where these molecules are being probed with a multitude of techniques across many timescales. New approaches to time-resolved crystallography are allowing simple “movies” to be taken of proteins in action, and new methods of mapping the variations in cryo-electron microscopy are emerging to reveal a more complete description of life’s machines. The results of these new methods are aided in their dissemination by continual improvements in curation and distribution by the Protein Data Bank and their partners around the world.

Except at a temperature of absolute zero, which is impossible to achieve, molecules exhibit dynamic behavior. A scale from fast vibrational states to conformational changes to complete folding/unfolding events exist and can be observed in proteins and other biomacromolecules. Crystallography has celebrated over 100 years of visualizing the atomic structures of elements and compounds and about 60 years of doing the same for macromolecules and large biological complexes. More recently, cryo-electron microscopy has rocketed to the forefront of structure determination methods, also revealing atomic or near-atomic detail. These techniques, together with NMR have yielded almost 200,000 experimental determinations of the average positions of atoms in an astoundingly diverse set of proteins and nucleic acids and their complexes. Graphic artists’ renderings regularly grace the covers of journals. Perhaps because vision is one of the strongest senses of humans, we peruse these structures eagerly, hanging theories on them about how they work, defining atomic level mechanisms, and generating new hypothesis for further exploration.

However, these macromolecules are not static at their physiological temperatures. We can often observe experimentally variations in structure and also sometimes time-dependent changes, *i.e.*, dynamics. A disclaimer on use of the word dynamics should be given here. No one would argue with the idea that proteins with the same amino acid sequence exist in variety of states at some level of detail, and that at modest temperatures there are interconversions between these states, hence dynamic processes are taking place. Theories of induced fit (1) and conformational selection (2) require a rearrangement of atoms in some kind of dynamic process. In this review, a broad definition of protein dynamics is used, including kinetic changes in particular structures and changes in populations within distributions of conformational states (3). Not included here is another definition of protein dynamics that is more akin to protein turnover, with time-dependent changes in generation and degradation of pools of proteins.

Our understanding of relationships between structure, dynamics, and function has been multidisciplinary, comprising work of theorists, computationalists, and experimentalists from physics, chemistry, and the biosciences. Thankfully there are some common mechanisms of communicating structure results in carefully defined ways. The value of scientific research is almost nothing if not shared with others; the communication of these atomic arrangements is paramount and yet publishing a list of numbers in a journal is not feasible beyond describing those in a modest-sized organic chemical, much less a protein. Protein crystallography did not exist before computers, at least not the “solving” part, and the value of storing the coordinates in electronic form seemed obvious. The idea to curate them and share them was not so obvious or even popular at first. Initially implemented at Brookhaven National Laboratory in 1971 (4, 5, 6) the Protein Data Bank (PDB) has come to be a positive example and a paradigm of community-based data sharing.

The original PDB was designed to hold and distribute one set of coordinates per structure. In crystallography, these would typically be results of a refinement that worked to minimize the differences between measured diffraction patterns and those calculated from the set of atomic coordinates. For NMR they would be either a representative single structure or an ensemble of models, selected as example structures that met stringent requirements for being commensurate with the measured data. For cryo-EM these coordinates would be interpreted from carefully averaged images of single particles. In any case, there is still a challenge to add dynamically changing coordinates to the PDB, while dynamic processes are seen to be increasingly important for our understanding of protein function.

## Myoglobin and adenylate kinase—two model systems for studying protein dynamics

Some examples of the quest for knowledge of protein dynamics from the authors’ own work and others are described below. These works illustrate some collective progress in observing protein dynamics and interpreting it in the context of biological function. The studies will be presented in order from small, fast dynamic events to larger, slower ones.

**Figure 1:**
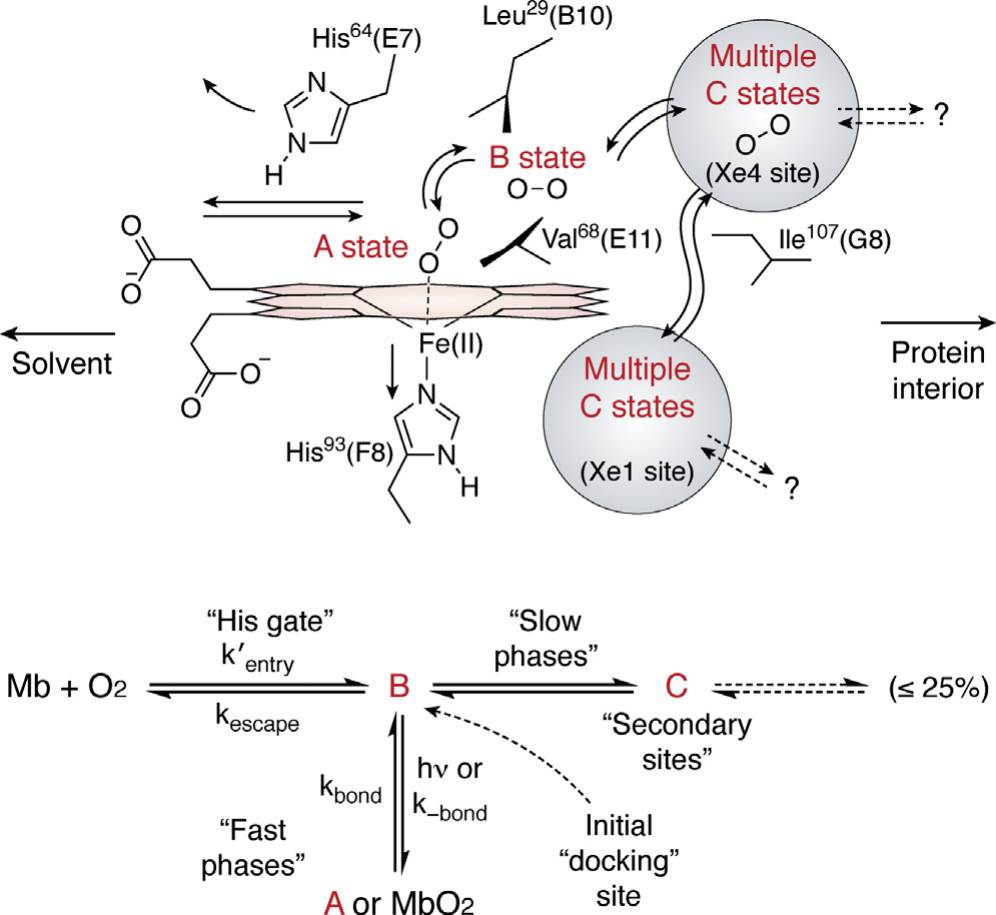
**Stereochemical interpretation of the side path scheme for geminate recombination (from Gemini, the sign of twins—referring to rebinding of the photodissociated molecule before diffusional escape) and bimolecular binding of O**_**2**_**from solvent.***Upper panel*, photodissociated ligands first move into the empty space toward the back of the distal pocket (state B). Some of the molecules move further into the protein toward the Xe4 and perhaps Xe1-binding sites (states C). O_2_ moves back from these more remote positions into state B and either rebinds to form MbO_2_ (state A) or escapes when the distal His64 gate is open. *Lower panel*, this reaction scheme is proposed, where k 1 = k bond, k 2 = k escape, and k 3 and k 4 equal the rates of movement into the secondary sites and back to the primary site or state B (9).

Myoglobin, a small oxygen-carrying protein, the first protein to have its three-dimensional structure elucidated (7), was also immediately recognized to need dynamic components for its biological function, as there was no open pathway for the oxygen to get into and out of the protein without some rearrangement (8). The shortest way in and out of the heme binding seemed to involve getting past the so-called distal histidine side chain, which in the crystal structures typically blocks the path (9) (Fig. 1). Myoglobin became a model system for all kinds of multidisciplinary studies in protein science, even being referred to as the “hydrogen atom” of biology by the prominent biophysicist, Hans Frauenfelder and colleagues (10).

The study of the dynamics of myoglobin is aided by the presence of an iron-containing heme cofactor that binds oxygen and other small compounds, such as the toxic molecule carbon monoxide. This group is also what makes myoglobin and hemoglobin red colored, giving a visible spectroscopic handle. The heme group thus provides opportunities for various spectroscopic experiments to probe the details of ligand binding and unbinding and sources of specificity for the biologically required oxygen.

Even before the recent development of time-resolved crystallography experiments, a huge body of work existed to describe the dynamic binding and unbinding of small gaseous ligands to myoglobin and hemoglobin. In myoglobin, a flash of laser light can be used to rapidly break the covalent bond between the carbon of CO and the iron atom of the heme, termed photodissociation (11). Hans Frauenfelder and his collaborators in a series of papers experimentally identified the number and kinds of energy barriers that the CO had to overcome to rebind to the iron atom. Heterogeneity in the conformations of the protein manifested as nonexponential rebinding events, in which different substates of the system have different intrinsic rates and do not follow one classical exponential process. Later work (12, 13) showed relaxation of the protein is crucial for the escape of the ligand from the pocket, as was suggested earlier by Friedman and colleagues (14), where linkages were shown to exist in the related protein hemoglobin, between the protein conformations and events at the iron atom and the heme group.

Vibrational (infrared) spectroscopy has been particularly useful for showing the interplay between conformational dynamics and function in myoglobin. Because of its triple bond, the stretching frequency of CO is shifted to a region not complicated by other signals. The CO then becomes a sensitive monitor of properties of the surrounding protein. In a series of studies, Nienhaus and coworkers observed the effect of the protein and its different effects on CO, including states shortly after it is unbound from the iron atom, and when trapped in preformed cavities inside the protein matrix (15, 16). Myoglobin was also shown to “relax” to a new conformation after photolysis that affects the kinetics of its rebinding (17). Furthermore, mutagenesis has been extensively used to perturb the structure and dynamics of myoglobin and test models of its dynamic behavior (18, 19, 20). In one study the stretching frequencies of the CO and the iron-C bonds were correlated with oxygen and CO binding affinities through an electrostatic process (21). And other studies probed the need for dynamic components of ligand entry and escape including removal of the distal histidine gate (19), or opening the gate with lowered pH (22) or propping the gate open with a bound ligand, not unlike leaving a trail as in Ariadne’s thread (23).

Taken together, these results suggest that not only chemical bond formation and electrostatics (24) but also transient passageways within the protein matrix are determinates of the rates of rebinding of the iron to (or release from) the protein. These transient kinetic processes may serve as some sort of kinetic proofreading that supports more preferential binding of oxygen (21).

Looking deeper into the quantum mechanical and vibrational world of myoglobin and the biochemistry of its iron, Falahati *et al.* (25) have described a dynamic process during ligand dissociation involving oscillatory dynamics of the iron in the heme during a spin transition that breaks the symmetry of the system, encourages the transfer of an electron from the porphyrin to the iron, and retards rebinding of the ligand to the iron. This same spin transition forces the movement of the iron out of the plane of the heme and is part of the description of the mechanism of cooperativity in hemoglobin (26, 27).

The overall dynamics of the entire myoglobin molecule have also been studied by a variety of methods, including X-ray crystallography, neutron scattering, and molecular dynamics (MD) simulations. Comparisons of the dynamics in different crystal forms, where the amplitudes of small harmonic displacements can be fit to the diffraction data, showed a consistent pattern of overall motions, once the crystal packing effects were taken into account (28, 29). In another study, elastic, quasi-elastic, and inelastic components of neutron scattering were measured and compared with calculations from an MD trajectory, showing good correspondence and supporting an atomic model describing atomic displacements on the 0.3- to 100-picosecond time scale (30). All of the results describe a situation where some parts of the protein are more mobile, namely, the connection between the C and D helices (CD corner) and the N and C termini. The dynamics of the CD corner, particularly at position 46, allow coupled positioning of the distal histidine for hydrogen bonding to oxygen and to the opening of the histidine gate (31).

The role of the solvent in defining protein dynamics in myoglobin has also been explored to show aspects of the water dynamics (32) and slaving of the protein conformational transitions by the solvent (33).

**Figure 2:**
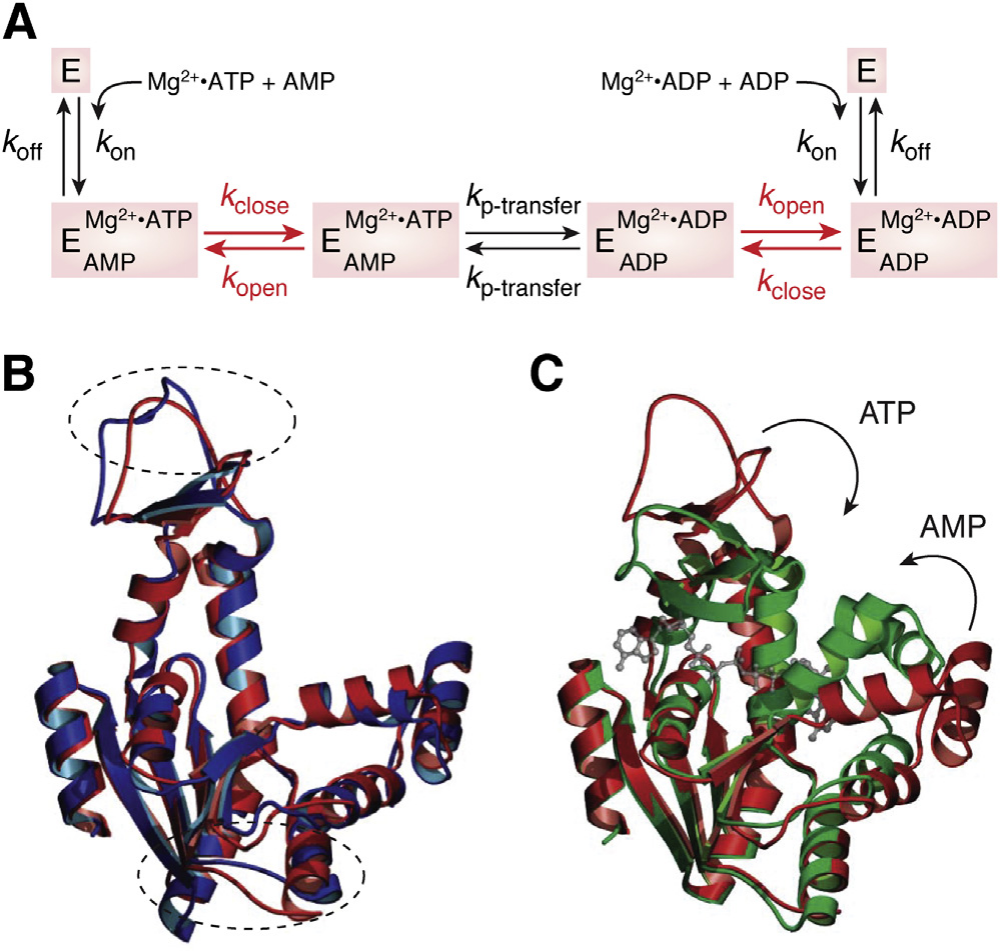
**The enzyme adenylate kinase undergoes large, dynamic conformational changes during its catalytic cycle.***A*, proposed reaction scheme for the enzyme adenylate kinase (E) including the steps of substrate binding (*k*_on_), lid closing (*k*_close_), phosphotransfer (*k*_p-transfer_), lid opening (*k*_open_), and substrate dissociation (*k*_off_). *B*, superposition of molecule A of apo *Aquifex* (*red*) with apo *E. coli* (*blue*) Adk reveals only small changes in the overall structure between the homologues, as indicated by the *dashed ovals*. *C*, superposition of apo *Aquifex* Adk (*red*) and *Aquifex* Adk in complex (*green*) with the substrate analog Zn^2+.^Ap5A (shown as *ball* and *stick* in *gray*) demonstrates the closure of the ATP and AMP lids on substrate binding. Figure from (40).

Another commonly studied model system for protein dynamics is adenylate kinase, a small enzyme that catalyzes the reversible transfer of a phosphate group from ADP and AMP to maintain an equilibrium among ADP, AMP, and ATP. The motions are dramatic, including the opening and closing of a “lid” and a “flap” that cover the ATP- and AMP-binding sites, respectively (Fig. 2). At present there are over 1200 papers with adenylate kinase in the title *via* PubMed. Enzymology (34, 35), genetics (36), crystallography (37, 38, 39, 40), solution scattering (41), NMR (42, 43), MD simulations (44, 45), thermodynamics and unfolding (46, 47), single molecule studies (48, 49), hydrogen exchange mass spectrometry (43), various spectroscopies, mutagenesis (50, 51), evolution (52, 53), phylogeny (54), and bioinformatics (55), and others have all been employed to gain insight regarding the connections between structure, dynamics, and function. There is not a single model for a pathway commensurate with all the studies. Recent contributors to work on adenylate kinase make regular use energy landscape theory in their interpretations (56, 57, 58, 59).

The protein is widely studied by structural methods. There are no fewer than 115 PDB entries with adenylate kinase in the title, representing 27 species and multiple states of the protein with and without bound ligands and metals, bound inhibitors, and occasionally ensembles of structures that represent interconverting states of the enzyme. These coordinates have been used to infer dynamic transitions (60) and catch glimpses of intermediates (38, 40) and examine ensembles from NMR (61) and crystallography (Bae and Phillips, unpublished). They have also been mapped to energy landscapes created from MD simulations (44, 45, 62). The availability of coordinates *via* PDB entries has been essential to the interpretation of experimental and theoretical studies, enabled MD simulations, and generally informed us about structure–dynamics–function relationships in adenylate kinase as well as many other protein systems.

One postulate is that the large-scale motions are not random but follow directions that promote the needed chemistry for catalysis (40). However, the time scale of the large amplitude lid opening of adenylate kinase is too long to be directly relevant to the actual catalytic step (63), leaving the question open whether faster short-range motions couple to a transition state in enzymes. An interesting correlation between temperature, dynamics, and enzymatic function led to the corresponding states hypothesis, wherein organisms adapted to different temperatures have enzymes, including adenylate kinase, that tune their degree of dynamic behavior to be similar despite operation at different temperatures (51). Whether the (needed) dynamics are part of a required process in molecular recognition/specificity of binding or whether they are coupled directly to the formation of a transition state by orbital steering is still an open question. Simulations suggest enzyme protein dynamics can be satisfactorily described as equilibrium fluctuations along the reaction coordinate, which is in line with a high degree of preorganization being a feature of the enzyme, and promoting quantum mechanical tunneling (64).

Recently, a crystal structure was obtained of a ternary complex with ATP, AMP, and Mg^2+^ allowing (quantum mechanics/molecular mechanics) calculations that show a direct dynamic role for some of the residues in the active site, dynamically changing the hydrogen bond arrangements of side chains with the substrates and thus presumably helping cross energy barriers for catalysis (65). This is an important step forward in connecting dynamics to catalysis.

The consensus seems to that early notions of simple openings and closings of segments surrounding the active site to allow access were overly simplistic and that aspects of conformational selection, local melting, or cracking with allosteric effects and orbital steering need to be part of the defined mechanism. Some studies suggest an ordering of substrate binding, but the original enzymatic studies implied a random bisubstrate biproduct (bi-bi) mechanism, at least for the rabbit muscle protein, for which either substrate can bind first and either product can leave first (34). Adenylate kinase from many different species have been studied, and it might not be true that they all have exactly the same mechanisms. There is even some evidence that different pathways of lid opening are taken depending on the temperature, with the flap opening being more entropy driven (62). It does seem to be clear that there is a central core that stabilizes the overall folding of the protein and that the lid and the flap and the side chains near the active site have effects on catalytic rates, requiring rather dramatic dynamic processes. Clearly a detailed pathway describing the motions of the lid and flap and their roles would be desirable.

## Making movies from experiment

Just as Muybridge is credited with producing the first “movie,” a running horse displayed as a time series of snapshots (66), the development of intense appropriately pulsed X-ray beams and ways of initiating events in protein crystals has allowed us to move beyond snapshots to primitive movies.

**Figure 3:**
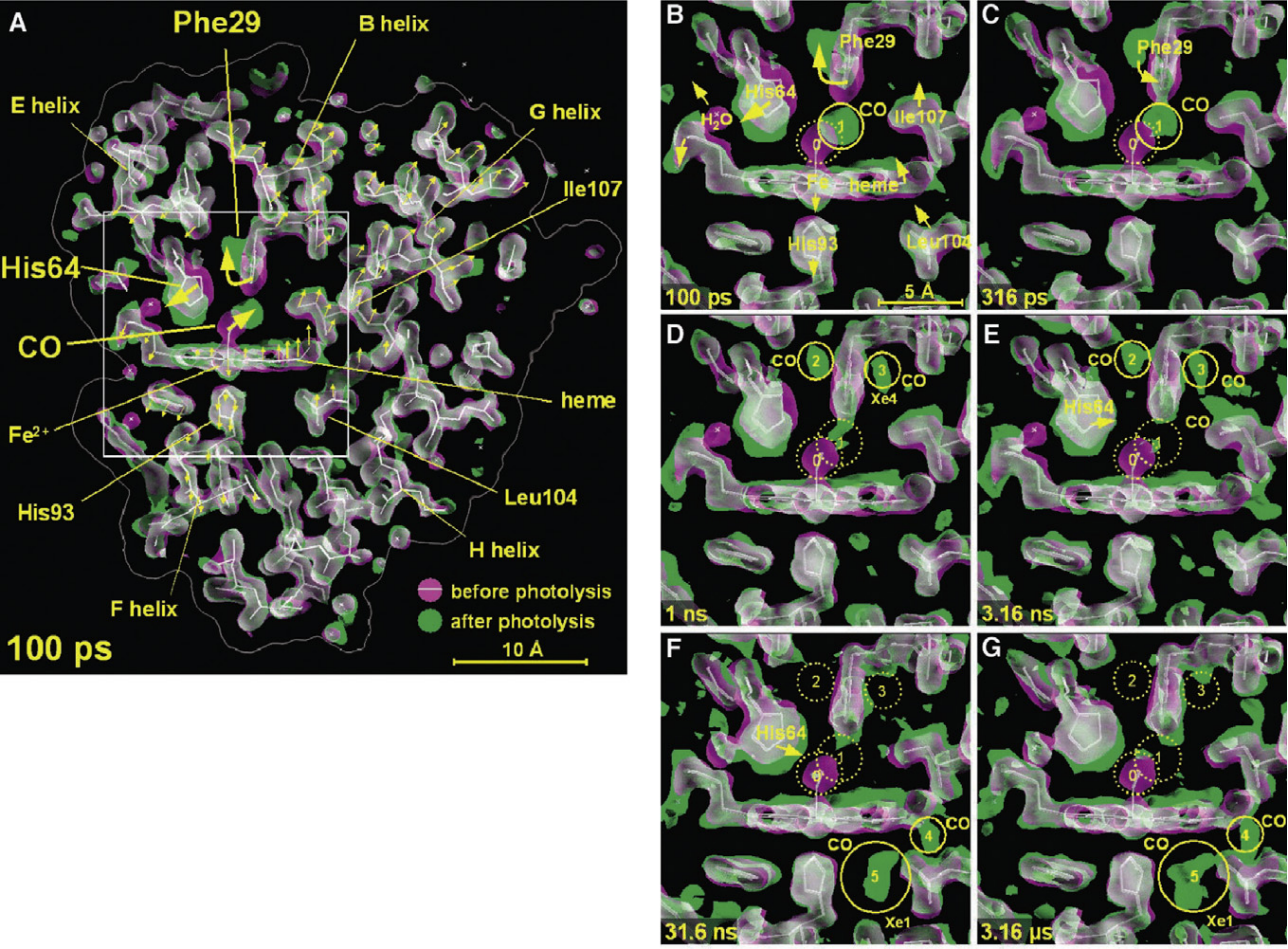
**Images of changes in the electron density maps of myoglobin after photodissociation of carbon monoxide with a laser pulse.** The ligand and protein begin (overview panel *A*) in the conformations represented by the *purple* density and migrate to the *green* states as time elapses (detail panels *B*-*G*), showing the dynamics of the process. The CO moves up and to the left, diffusing into the protein matrix. From (68).

Synchrotron radiation coupled with the reversibility of the binding of small gasses such as molecular oxygen and carbon monoxide (67) allowed for real measurements of dynamic events on the nanosecond time scale, allowing time-resolved crystallography. The work was extended to subnanosecond (68) and longer millisecond (69) time resolutions and the refinement of atomic coordinates allowed animations of the departure of the CO from the heme-binding site (see video under supplemental information (70)). After initial photolysis the carbon monoxide moves to a docking site, causing rearrangements in the pocket residues, the coordinate changes of which can be seen as a function of time. These include rotations of the heme-pocket phenylalanine concomitant with movement of the distal histidine toward the solvent, potentially allowing carbon monoxide movement in and out of the protein and proximal displacement of the heme iron (70) (Fig. 3).

The advent of the free electron laser has revolutionized the study of protein dynamics (71), allowing for the collection of complete three-dimensional data sets with various time delays after initiation of a protein’s functional cycle. A steady stream of very small crystals is streamed into the pulsing X-ray laser and some fraction of the crystals are struck, producing thousands of diffraction images of randomly oriented crystals. The injection of a multitude of small crystals is referred to as serial femtosecond crystallography (SFX). These images can be processed to give near-atomic resolution electron density maps of the average structure in the illuminated volume. When some reaction is triggered just before striking the crystals with the X-ray pulse, one can perform time-resolved SFX with subpicosecond time resolution (72), and including confirmation and extension of earlier picosecond work on myoglobin (73). Because the crystallography experiment averages over many repeating unit cells in the crystal, one observes distinct states only as long as they are synchronized in the crystal (74).

Notable examples of time-resolved SFX include light-induced transitions in photoreceptors (75, 76, 77) and the enzymatic opening of the lactam ring of antibiotic compounds (78). Systems likely to be amenable to this approach must meet certain requirements: the processes to be observed must be triggerable (by mixing or stimulated by light), the crystals must preserve order during the dynamic processes, the protein must be made and crystallized in large amounts (depending on the serial sample delivery method), and the results must generally be interpreted as mixtures of states as the initial triggered synchronicity is eventually lost by stochastic events.

## Cryo-electron microscopy and protein dynamics

Cryo-electron microscopy is also contributing to our understanding of protein dynamics. Electron diffraction, tomography, and single-particle imaging all contribute to our understanding of dynamics. Conformations along a pathway can be trapped by fast freezing to image different states (with time resolution limited by freezing times on the millisecond time scale) or by reconstructing a time series through analysis of the ensemble of particle image by advanced dimensionality reduction methods such as iterative classification or manifold embedding methods (79, 80, 81, 82). These methods also provide a type of “movie” that informs us about the function motions in proteins. Examples include rotations of ribosomal subunits corresponding to stepping in translation (see the movie in the supplemental information from (81)) and the opening of the ryanodine receptor (see the movie in the supplemental information from (79)). In these analyses it was shown how one can convert numbers of appearances of different conformations of the single particles to energies by the Boltzmann equation and hence produce a low-dimensional energy landscape with putative functional pathways.

As a part of the Worldwide PDB (wwPDB), which comprises members Research Collaboratory for Structural Bioinformatics-PDB, PDBe (Europe) and PDBj (Japan), and Biological Magnetic Resonance Bank under the 2013 charter and will soon include PDBc (China) and PBDi (India), there is the electron microscopy data bank (EMDB) (83), currently holding about 12,000 maps, with about 2000 associated sets of interpreted atomic coordinates.

## Molecular dynamics simulations and normal mode analysis

MD simulations, ranging from quantum mechanical to atomic to coarse-grained bases provide trajectories of motion that follow the underlying definitions of their underlying force field descriptions. These may or may not have accurate definitions of time but typically result in large data sets of evolving sets of coordinates. There is not a well-developed depository to share these data as there is for experimental structure determinations (84, 85), but there are some specialized efforts to make MD data more available (see listing in (86)). It is true that the actual trajectories have stochastic components, so that while they are perhaps not as unique as experimental measurements their analysis should be able to be reproduced by others. For an example visualization, see a simulation of the SARS-CoV-2 spike protein starting from PDB entry 6XVV with added glycan chains https://www.youtube.com/watch?v=7AhQ19m2ok4 (87).

**Figure 4:**
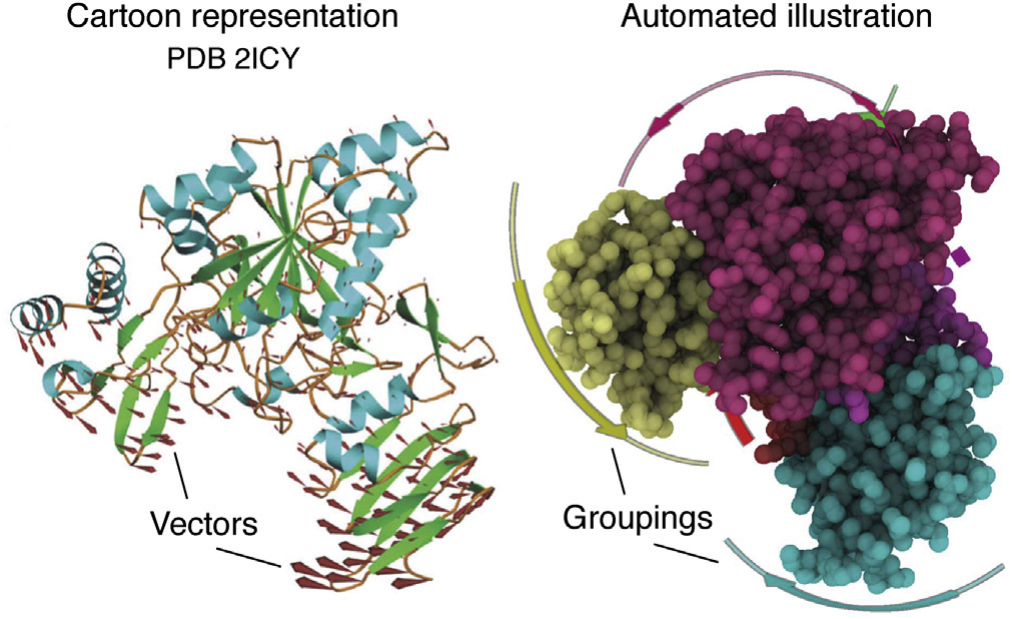
***Left*, vector plot illustration of a normal mode of a protein taken from** (135)**.***Right*, the same data, illustrated with an automatic approach that is also effective at showing the groupings (116).

To glean abstractions of the motions from an otherwise complicated MD trajectory, dimensionality reductions methods, such as principal component analysis (88), singular value composition (89), or other geometric methods (90), have been employed. If one is interested in the large-scale motions, these can be quite effective, but it is not guaranteed that the modes identified by the methods are the most important ones for biological function (Fig. 4).

There are other ways to predict and analyze the large-scale motions of proteins. Normal mode methods can serve to show the directions and relative amplitudes of motions for any set of coordinates, given their positions and the forces or potential energy functions describing their interactions. These models describe harmonic motions relative to the equilibrium positions of the atoms. The larger-amplitude modes seem more likely to be relevant in the formulation of mechanistic descriptions, but small-scale modes could also couple to the function of the protein. These methods can use the same atomic force fields used in MD or simplified models (91, 92). These models are very effective in matching the relatively small atomic displacements (B-factors) seen in protein crystal structures (93), especially when the packing of neighbors in the crystal are included (94). There are many cases where the large-amplitude modes match known features of a macromolecular system, (*e.g.*, see (95)). Easy ways to calculate these large-amplitude modes have been made available *via* web servers (96, 97). And a database of protein motions has been created (98, 99).

## Larger-scale molecular machines—motility and replication

When the function of a molecule to is physically translate or transport objects over some distance, it involved mechanical work and the required protein dynamics are quite dramatic. The manifestations of these protein motions extend to scales that can be seen by single molecule experiments (100, 101, 102), electron tomography (103), and light microscopy (104). Through a series of studies at resolutions from near atomic to macroscopic, the contraction of muscle fibers, the “walks” of dynein, kinesin (105), and myosin (106) are being elucidated in great detail. These can be thought of as dynamic Brownian machines (81), using ATP hydrolysis or other sources of energy to bias the direction and perform useful work. The concept of a thermal ratchet for rectifying Brownian motions is useful for many proteins that involve physical transport (107, 108, 109). This concept can be extended to include a broader range of protein functions by thinking in terms of kinetic asymmetry instead of spatial asymmetry providing a mechanism by which chemical free energy released by catalysis can drive molecular adaptation and self-assembly as well (110). Artists’ renderings of the machinery in action that drives muscle contraction or motility can be found in the fascinating YouTube videos linked here: https://www.youtube.com/watch?v=oHDRIwRZRVI https://www.youtube.com/watch?v=y-uuk4Pr2i8.

**Figure 5:**
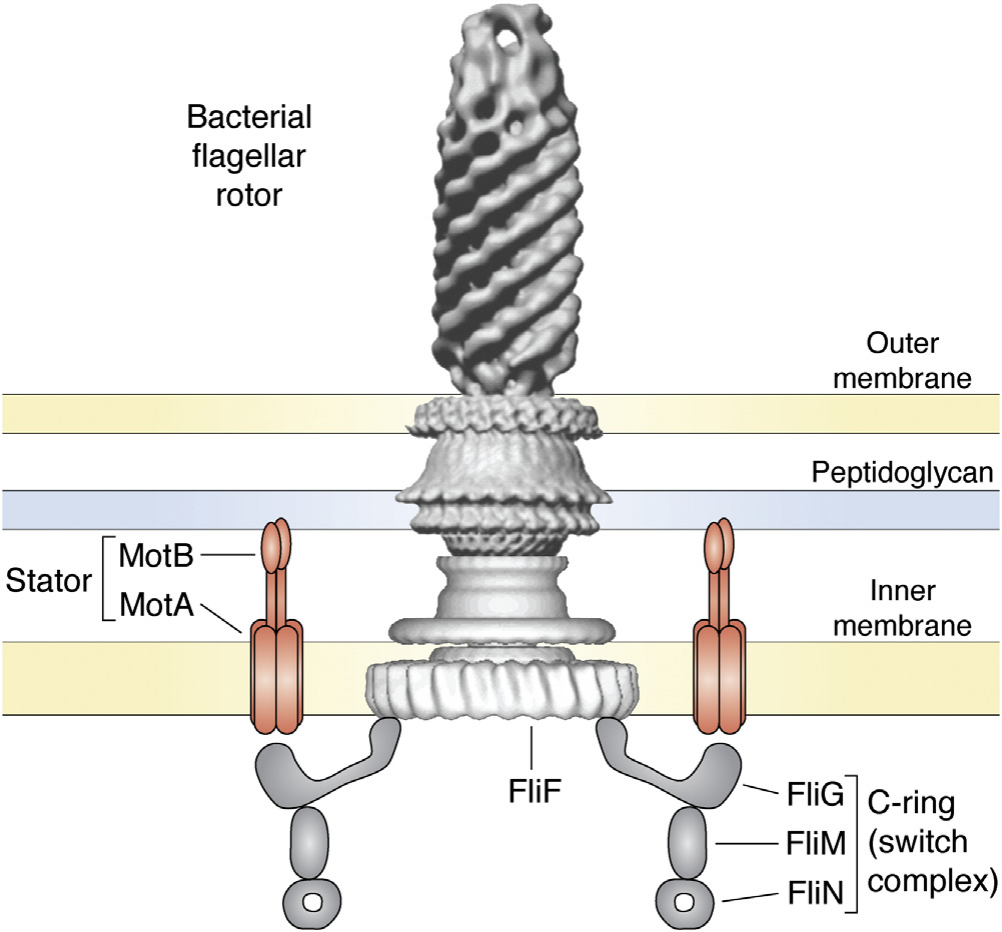
**The protein components of the bacterial flagellar rotor self-assemble to form a rotating “motor,” twisting a propeller attached to top of the motor axis** (111)**.** It can be turned on and off by protein dynamics in switching systems to allow taxis toward food or light or away from toxic elements. Figure from (136).

Another fascinating protein system is the flagellar motor. Similar to being driven by electric motors, these rotating propellers use the energy from ATP hydrolysis to turn flagella and move the organism forward. We have a good picture of the architecture of the motor through light and electron microscopy (111) (Fig. 5). An artist’s rendering of the parts diagram and the motor in action can be found in this video: https://www.youtube.com/watch?v=cwDRZGj2nnY.

The big event of life on earth is the replication of DNA, allowing organisms to reproduce using precise molecular blueprints. The molecular machines that replicate DNA (DNA-dependent DNA polymerases) or translate DNA to RNA (DNA-dependent RNA polymerases) are highly sophisticated and dynamic protein assemblies. Through X-ray crystallography and cryo-electron microscopy the sequence of events required for these fundamental functions in biology have resulted in well-illustrated animation videos summarizing person-decades of research on the individual subunits and coordinated activities.

**Figure 6:**
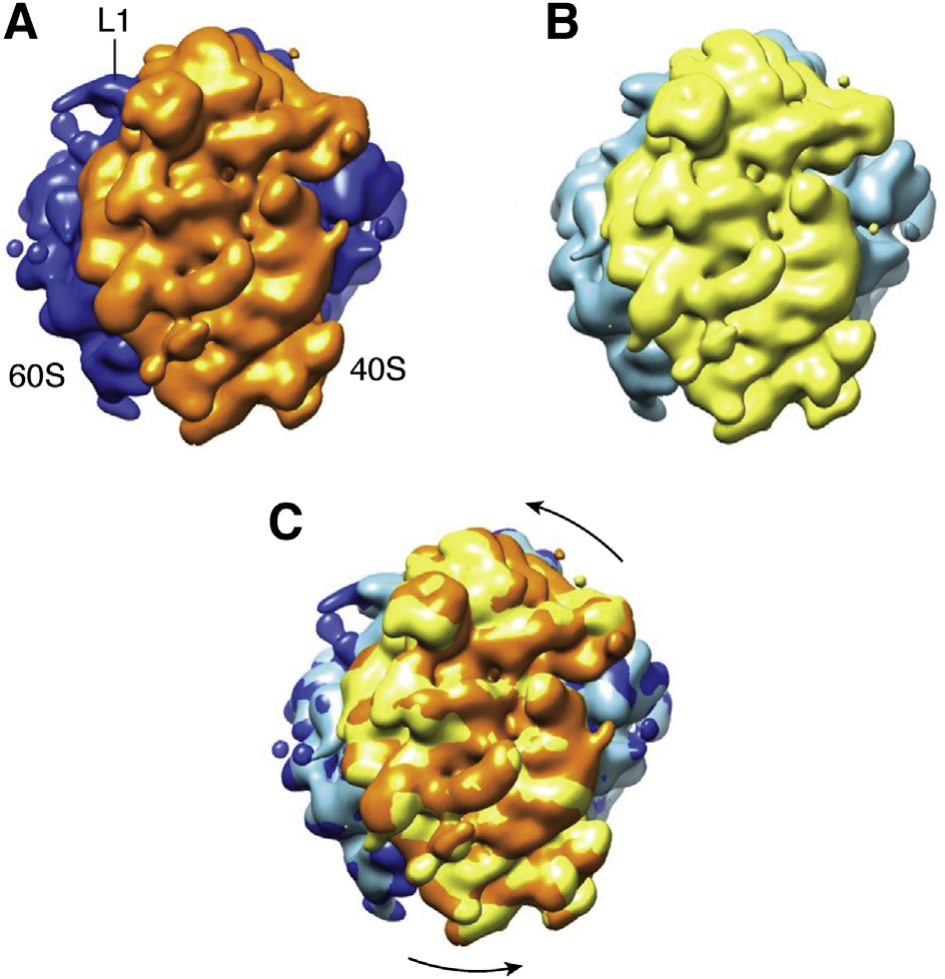
**Relative rotation of the ribosomal subunits during the process of protein translation.** The top subunit rotates slightly counterclockwise from the *orange* (*A*) to the *yellow* (*B*) position during a cycle. The superposition is shown in (*C*). Figure from (81).

The same is true for the translation step from mRNA to protein. The ribosome, a complex assembly of both protein and nucleic acids, undergoes a sequence of steps that move along the RNA strand, reading the genetic code and adding the appropriate amino acids (Fig. 6). Recently, an energy landscape approach using manifold embedding has been used to extract a sequence of conformations from a set of thousands of single-particle cryo-EM images to yield a movie of a cycling ribosome that seems to correspond well with biochemical steps known to occur during the translation process (81).

Single molecule studies of protein dynamics yield a picture of function where every molecule behaves differently but on average suitable function is achieved (112). Although generalizations are clearly helpful, it may be true that no two proteins have ever had exactly the same structure.† Heterogeneity exists in the populations of enzymes and other molecular machines either before their native folding is complete (114) or after.

**Figure 7:**
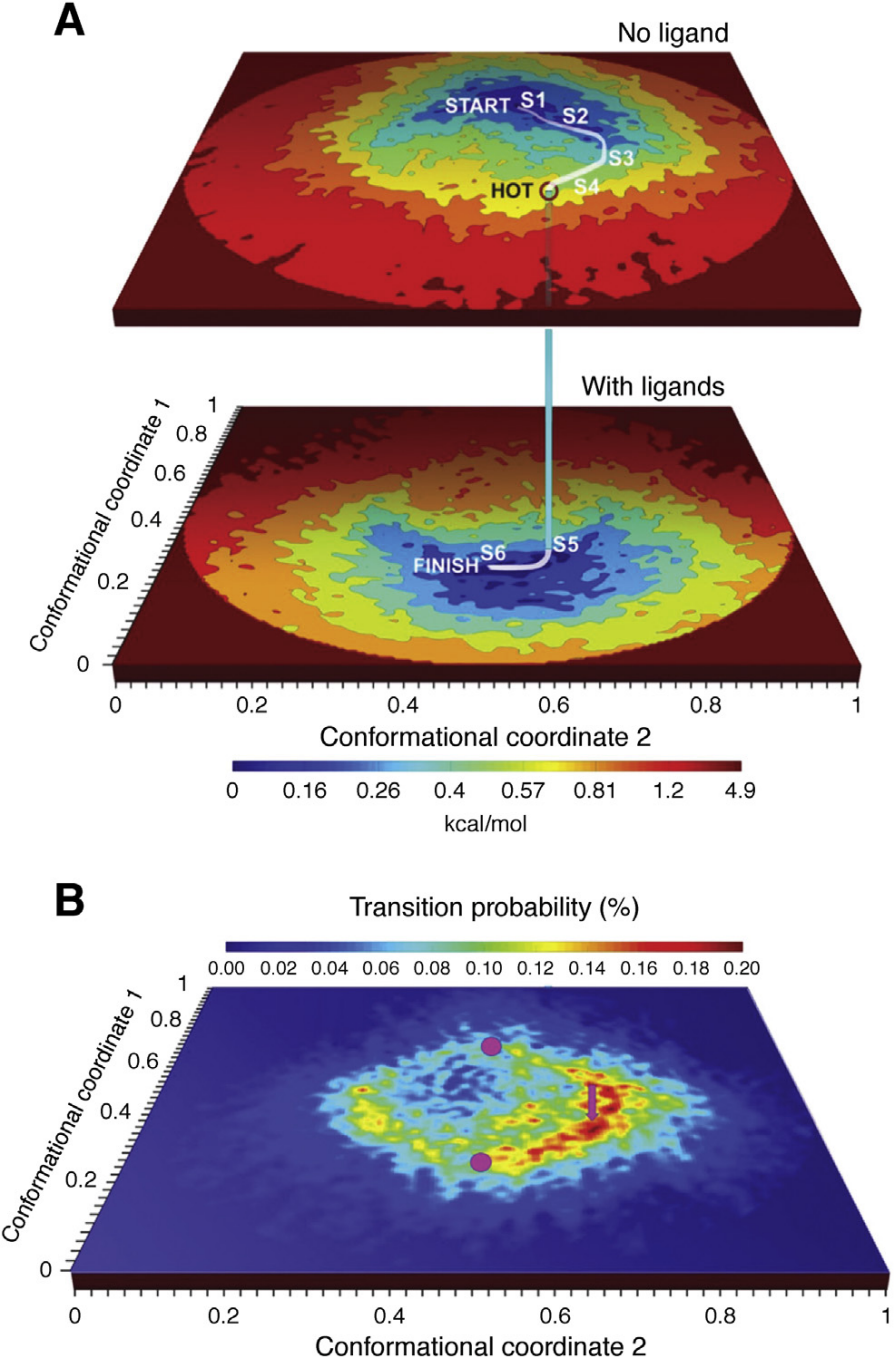
**Energy landscapeanalysisof the ryanodine receptor channel based on observation of single particles from cryo-EM.** Energy landscapes from of the apo (*closed*) state (*A*, *top*) and ligand-bound (*open*) state (*A*, *bottom*). The curved path shows one probable path from the closed to open states. Shown in *B* is the probability map for the transition from closed to open states (79).

The concept of using changing energy landscapes offers a more complete description of the connection between structure, dynamics, and functions as it can incorporate both single molecule and ensemble measurements into a holistic framework. The Boltzmann equation can relate free energy (or at least some chemical potential) to a distribution of states. The likelihood of a protein conformational change by say, ligand binding, would be described by a joint transition probability calculated from the density of occupied states from the bound and unbound energy landscapes (79, 82) (Fig. 7).

## Role of dynamics in function

“What I cannot create, I do not understand.” Feynman may have been talking about derivations and theory, but it still may apply here. We can fold proteins, but we still cannot create useful enzymes. What are we missing? Antibodies can force a compound into a near transition state, but the catalytic rates are poor. Are we missing some dynamic component that steers orbitals toward a reaction (115)?

One of the difficulties in connecting dynamics to structure–function relationships is the sheer complexity and the lack of tools to distill a raw structure or trajectory to something useful. Frauenfelder spoke of functionally important motions (nicknamed “fims”), but there can also be biologically unimportant motions (coined “bums” by A.R. Crofts at about the same time). How does one tell the difference? Narratives where data on the dynamics are woven with other kinds of knowledge to yield a consistent and physically reasonable model are the most satisfying. The resulting hypotheses can then be tested by changing the temperature or changing the dynamic profile of the protein by mutagenesis or chemical perturbation. Tools for distilling protein dynamics into patterns that humans can parse for structure function relationships are scant. Watching (or evaluating by calculation) an MD trajectory for rare events that might be relevant is common but does not constitute an experimental verification. Visualization of the large-amplitude normal modes can be helpful if the fims are captured in the low-order modes (97, 99, 116), but this is not a given. Dimensionality reduction methods can also be used to explore the landscape of possible functional connections (89). Kinetic trapping at cryogenic temperatures is possible now by electron microscopy, crystallography, and other spectroscopic methods. Physiological temperature, single particle imaging methods with near-atomic resolution offer real promise in imaging structure and dynamics of proteins but are not yet feasible at high resolution (117).

Analysis of the trajectories and connections to function are increasingly computable. One example method seeks to identify barriers along the most probable transition path for a protein functional event, yielding parameters that can be compared with experimental values, including analysis of trends that result from mutational perturbations at the key sites (118, 119). These approaches, starting simply from PDB coordinates, may at least reveal the energy coupling between side-chain interactions that are on the functional rate-limiting path(s).

From the very first crystal structure of myoglobin, where it was noticed that the oxygen could not get into or out of the protein without dynamic rearrangement, it seems clear that dynamic descriptions will necessarily be a part of the discourse on etiology of protein structure–function relationships. It is cleaner to build a narrative using a single prototypical trajectory, not unlike a Rube-Goldberg machine, but we know from single molecule enzyme kinetics that each individual protein molecule has its own (stochastic) rate. Since we are always dealing with a fluctuating ensemble of proteins with dynamic interconversions of states, we argue that a more sophisticated approach would be to speak in terms of movement on, and the shifting of, energy landscapes during a protein’s functional cycle.

## Data management and sharing of dynamics results

The ways protein dynamics data are deposited at the PDB are varied and sometimes creative rather than standardized. In the case of a paper describing a method of extracting time points from the study of thaumatin and radiation damage, only the first time point was deposited (120), with stroboscopic figures of density map changes over time presented in the article. In another study of bacteriophytochrome, the raw data comprising different time points were deposited, and a basic set of four intermediate coordinate files that were used to fit the diffraction data were deposited (121). The validation does not work in this case as any fit would require a linear combination of states. Refinement of coordinates at each time step sometimes occurs, but capture of the data quality does not always make it into the PDB entry in standard ways (122); sometimes only some of the time points are refined and deposited with others shown as difference maps in the paper (123), and in cryo-EM only some maps deposited (79). With more effort, some authors deposited a large number of time points as coordinate sets, as for a study on bacteriorhodopsin with 14 related depositions (124). All the above authors should be applauded for sharing their data in some way. Not all do.

In the cases where explicit coordinates are determined for each time point, then deposition is more standard. The title just needs to indicate a time point in a series, then the user can produce “movies” and analyze the results directly.

The data from time-resolved experiments can be and are sometimes, but not always, provided to the PDB for deposition and sharing. Sometimes results are published not as sets of coordinates but in the form of difference maps, which requires some “creativity” in a PDB deposition. The mmCIF query system offers ways to include these data, including multiple experimental data sets in one deposition. One just needs a control structure, like an apo-state or a dark, nonilluminated state to anchor the deposition with a set of coordinates, then results from the time points can be attached to this entry. Validation remains a problem for the nonstandard ways of sharing the data, but at least it is available to the public. In the future, single particle coherent X-ray diffraction images may yield new structural data (125), and these studies can and should also be shared in easily accessible digital form (126).

Sharing of actual structural, dynamic, and functional data will be increasingly important as artificial intelligence and machine learning efforts work to integrate heterogeneous kinds of information into knowledge of the behavior of proteins and other macromolecular machines. For data sharing and management of activities related to protein dynamics, there are regular workshops where community members assemble to discuss and propose better ways of incorporating new kinds of data and structure determination in general (127). Spectroscopic data other than that from NMR are highly relevant to dynamics and are included in these discussions, including new standards for deposition of models obtained from integrative or hybrid modeling (127). The mmCIF query system (128, 129) now implemented by the PDB is quite extensible and should be able to handle new data types related to dynamics. A continual effort will be needed to encourage authors to properly describe and share results. Journal editors and funding agencies also need to insist on the sharing of data to help ensure the integrity and lasting value of the scientific efforts. The PDB also should continue to lead the community to develop standards for curation of dynamics data as they have successfully done for static structures.

## Data to knowledge: Dynamics concepts

The way of presenting interpretations of data related to dynamics is a critical part of the curation and discourse components of the scientific work. Presenting dynamic data on the printed page is challenging (130) but relatively easy with video. Stroboscopic sequences like those in the Muybridge movie or in Figure 3 are sometimes effective but quite busy if the data are in a three-dimensional form, as most protein representations are. So-called porcupine plots (Fig. 4) are possible in VMD (131), Chimera (132), or PyMOL (133), as are superposed members of ensembles, which are sometimes effective but the time order of the structures is lost. Most journals are fine with providing links to video that open in another application but few allow embedded video. There is a viewer called LENS, introduced by *eLife* and then picked up by six HighWire journals (including the *Journal of Biological Chemistry*) for a pilot, which allows simultaneous reading of the article and watching video material. Example trajectories of dynamic functions of proteins are quite compelling, especially to visual learners. The PDB has MOL∗ (134), a powerful visualizer for interacting with static structures, and some simple dynamics support.

More creativity is needed to turn dynamics data into knowledge about the underlying mechanisms of actions of proteins. Perhaps what we are missing in our ability to design enzymes *de novo* is a lack of ways of appreciating the dynamic components.

## Conflict of interest

The authors declare that they have no conflicts of interest with the contents of this article.

## Footnotes

† As a variation of the Levinthal paradox (113) it is easy to show that, even if all the backbone atom locations are in identical positions, even side chain rotational and vibrational quantum states are astronomical in their possibilities. Hence the question is only how broad a distribution of protein states is.
